# Circadian clocks and breast cancer

**DOI:** 10.1186/s13058-016-0743-z

**Published:** 2016-09-02

**Authors:** Victoria Blakeman, Jack L. Williams, Qing-Jun Meng, Charles H. Streuli

**Affiliations:** Faculty of Biology, Medicine and Health, and Wellcome Trust Centre for Cell-Matrix Research, University of Manchester, Oxford Road, Manchester, M13 9PT UK

## Abstract

Circadian clocks respond to environmental time cues to coordinate 24-hour oscillations in almost every tissue of the body. In the breast, circadian clocks regulate the rhythmic expression of numerous genes. Disrupted expression of circadian genes can alter breast biology and may promote cancer. Here we overview circadian mechanisms, and the connection between the molecular clock and breast biology. We describe how disruption of circadian genes contributes to cancer via multiple mechanisms, and link this to increased tumour risk in women who work irregular shift patterns. Understanding the influence of circadian rhythms on breast cancer could lead to more efficacious therapies, reformed public health policy and improved patient outcome.

## Background

The global impact of breast cancer is large and growing. With 1.67 million cases reported in 2012, it is the second most commonly diagnosed cancer worldwide [1, 2]. The incidence of the disease is much higher in the developed world, with four times as many cases in Western Europe as in Middle Africa and Eastern Asia. This suggests that aspects of a modern western lifestyle may influence the onset and progression of breast cancer. One possibility is a disruption to our internal body clocks, known as circadian clocks [3].

Intrinsic circadian clocks are driven by environmental time cues such as the natural day/night cycle. Our bodies translate timing cues into molecular oscillations within individual cells, which then drive 24-hour rhythms in cellular processes in almost every tissue in the body [4–6]. These cell-autonomous molecular oscillators make up the body’s internal timing system, and are synchronized by the master pacemaker, the suprachiasmatic nucleus (SCN) [7]. However, circadian clocks can become perturbed through irregular shift work, through repeated bouts of jet lag and during ageing. Weakened or damaged circadian clocks alter the susceptibility to certain diseases and directly drive others.

One of the processes regulated by the circadian clock is the cell cycle. Disruption of circadian rhythms can therefore be associated with abnormal cell divisions that occur in cancer [8]. Indeed, there are links between altered circadian clocks and tumorigenesis in metastatic colorectal cancer, osteosarcoma, pancreatic adenocarcinoma and, most notably, breast cancer [8].

The influence of altered circadian rhythm on breast cancer was first noted in the 1960s [9]. Since then, it has become clear that circadian disruption interrupts the complex multi-step molecular mechanisms underpinning breast cancer [1]. The indication that tumorigenesis is linked to circadian rhythms suggests that manipulating those rhythms might be a remedial approach for treating cancer. This could, for example, lead to more efficacious therapies, novel adjuvant strategies and, ultimately, improved breast cancer outcome [10].

In this review, we discuss the current understanding of links between circadian disruption and breast cancer risk.

## The SCN as a central pacemaker

Located in the anterior hypothalamus, the SCN is the central pacemaker that coordinates circadian rhythms with the solar day [11]. The bilateral SCN receives innervation directly from the retina via the retinohypothalamic tract. The majority of its ~20,000 densely packed neurons are ‘pacemaker cells’, with each neuron containing its own oscillatory machinery capable of producing a prolonged and robust circadian rhythm even in ex-vivo culture [12].

Light is a dominant synchronizing time-giver. However as well as light/dark cycles, the SCN is also responsive to changes in rest/activity cycles [5]. Non-photic stimuli, such as neuroendocrine signals and feeding behaviour, can also influence SCN pacemaking [5]. The SCN uses several neural and endocrine outputs to synchronize clocks of many peripheral organs [6]. One of these is the hormone melatonin [13], which is released rhythmically at night and relays information to peripheral organs [14].

The SCN is not actually required for peripheral organs to generate their own rhythms. Rather, it acts more like the conductor of an orchestra, guiding each organ to oscillate in the ideal phase for that specific tissue [15].

## Circadian clock genes and the circadian cycle

The basic genetic regulation of the circadian clock is highly conserved across the animal kingdom. Most mammalian clock genes were first identified via mutagenic studies in fruit flies [16]. The core molecular clock generates oscillations in protein levels via a series of auto-regulatory transcriptional/translational feedback loops [17]. Several clock genes encode transcription factors, with the molecular clock driving rhythmic expression of downstream clock-controlled genes [18].

The major components involved in this cellular clock network include the transcriptional activators Circadian Locomotor Output Cycles Kaput (CLOCK) and Brain and muscle Arnt-like protein-1 (BMAL1). CLOCK has a paralogue, Neuronal PAS domain protein 2 (NPAS2), which compensates for loss of CLOCK in the SCN and peripheral oscillators [19, 20]. The other main components are Period (PER1 and PER2) and Cryptochrome (CRY1 and CRY2), which form the negative arm of a feedback loop [17]. Additional regulatory systems such as nuclear hormone receptors and epigenetic mechanisms have also been identified [21, 22].

Circadian gene expression is the result of a series of transcription and translation events. This leads to the expression of a different set of proteins that switches them off at the end the day. The process then begins again with timely removal of the repressors (PERs and CRYs).

**Fig. 1:**
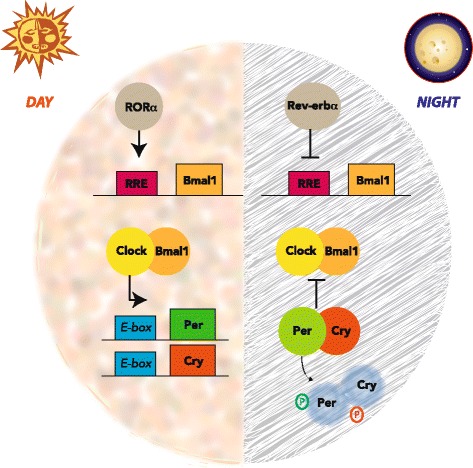
The core molecular circadian clock mechanism. Twenty-four-hour cellular rhythms are driven by an autoregulatory feedback loop. During the subjective day, RORα contributes to expression of *Bmal1* through its retinoic acid-related orphan receptor response element (RRE). The resulting CLOCK/BMAL1 complex activates transcription of the negative regulators, *Per* and *Cry*. By the evening *PER* and *CRY* levels accumulate to form a protein complex, which then becomes active as a CLOCK/BMAL1 inhibitor. During the subjective night, *REV-ERBα* suppresses expression of *Bmal1*, while the newly formed *PER*/*CRY* complex blocks CLOCK/BMAL1 activity, thereby preventing further transcription from the *Per* and *Cry* genes. During this time, the phosphorylated *PER*/*CRY* complex gradually degrades

At the beginning of the circadian day, *Bmal1* expression is driven by RORα, allowing the formation of CLOCK/BMAL1 heterodimers (Fig. 1). This transcription complex binds to CACGTG E-box sequences in the promoters of the *Per* and *Cry* genes, increasing their expression. The CLOCK/BMAL1 complex also increases expression of *Rev-erbα*, which suppresses *Bmal1* transcription [23]. The CLOCK/BMAL1-mediated increase in *Per* and *Cry* expression allows for the accumulation of PER in the cytosol, where it is phosphorylated by casein kinase 1ε and 1δ. Phosphorylated PER becomes ubiquitinated and is readily degraded, but CRY accumulation allows the formation of a stable PER/CRY/CK1 complex [7]. This complex inhibits the transcriptional capacity of CLOCK/BMAL1, preventing further expression of *Per* and *Cry*, and also *Rev-erbα*. Eventually phosphorylated PER and CRY are lost, de-repressing *Bmal1* transcription and allowing for higher levels of BMAL1 that start the next circadian day [18].

Collectively these cycles ensure that the circadian clock oscillates with a period of 24 hours, driven by the appropriate environmental time cues (Fig. 1).

## Peripheral circadian clocks

Peripheral tissues have their own intrinsic self-sustained circadian oscillations, but are dependent on the central clock and tissue-specific factors for synchronization. Local cellular oscillators regulate specific circadian programmes of gene expression that vary according to tissue function, with typically 5–10 % of all genes being transcribed rhythmically [24].

Clock target genes can be controlled directly by the CLOCK/BMAL1 complex, or indirectly via the circadian expression of transcription factors. In this way, the circadian clock confers a rhythmic aspect to a wide range of tissues. In the breast, for example, the expression of nearly 600 genes is controlled in a circadian manner.

The circadian regulation of gene expression impacts upon many cellular processes and complex behaviours. This is exemplified by SIRT1 and TIMELESS, proteins that independently intersect the clock and the cell cycle machines. For example, SIRT1 reduces proliferation by deacetylating β-catenin [25] and binding p53 [26], whereas TIMELESS advances the circadian clock in response to DNA damage [27, 28].

The circadian system is therefore well integrated with other physiologies. If the clock becomes disrupted, however, this can lead to disease [5].

## Clock genes in breast biology

There is growing evidence that circadian clock gene expression plays a role in breast biology. In mice, mutations of specific circadian genes disrupt behavioural and molecular rhythms. Such mutations also reveal the involvement of cellular clocks in the initiation of carcinogenesis.

Breast tissue contains a network of branched epithelial ducts surrounded by a basement membrane, outside which is a fibroblast-rich and adipose-rich stromal extracellular matrix [29]. Clocks have been discovered within breast epithelium using real-time bioluminescent imaging of mammary explants from PER2::Luciferase [30] mice. There are daily rhythmic variations in the expression of core clock proteins BMAL1 and PER2. Moreover, during the development of the gland from virgin through to full lactation, tissue isolated at the same time of day reveals that *Bmal1* and *Per1* mRNA levels increase in late pregnancy and lactation, while *Per2* falls [31, 32].

The levels of clock gene expression are also controlled by the breast tissue microenvironment. For example, we have found that a stiff extracellular matrix such as is found in ageing and cancer leads to suppression of core clock rhythms. Intriguingly, local stiffness of the breast stroma and extracellular matrix adjacent to breast epithelium has a major role in determining cancer outcome [33, 34]. The mechanisms linking the cell exterior to clock control are not yet known, but could involve the cytoskeleton and/or nuclear envelope proteins, one of which regulates transcription of the *Bmal1* gene [35].

The molecular clock is also crucial for regulating the survival of stem cells. Indeed the self-renewal capacity of mammary progenitor stem cells becomes compromised in mice that have a defective CLOCK/BMAL1 complex, revealing a circadian influence on breast function [36]. Studies in other tissues have demonstrated similar links between the circadian clock and stem cells. For example, undifferentiated neural stem cell cultures from BMAL1^−/−^ mice have an increased propensity to differentiate into glia over neurons [37]. In addition, circadian oscillations in the release of hematopoietic stem cells (HSCs), through chemokine *Cxcl12* and GSK3β, could result in a coordinated release of cells and thereby repopulation of the bone marrow stem cell niche [38].

Clock genes are expressed in normal breast. However, their levels are variable and controlled both by the cellular microenvironment and by the developmental stage of the tissue. Disruption of clock gene expression can increase breast cancer risk (Table 1).

**Table 1:** Genetic associations between circadian clocks and breast cancer

Mutation/SNP	Possible mechanism	Phenotype
CLOCK		Self-renewal capacity of mammary progenitor cells becomes compromised (our unpublished data)
Hypermethylation of *Clock* promoter	Mediates CCL5 expression	Reduced breast cancer risk [36]
NPAS2 Ala394Thr SNP	Altered NPAS2 protein structure	Increased breast cancer risk [55]
*Per1* deficient	Alters expression of checkpoint proteins ATM and Chk2	Increased proliferation [41]
*Per1* overexpression	Impairs p53 leading to decreased apoptosis, deregulation of c-*myc*/*CyclinD1*/*Gadd45*	Reduces proliferation in colon, lung and breast cancer cell lines [41]
*Per2* deficient	Increases OCT1 binding to EMT genes *Slug*, *Snail* and *Twist1*	Higher tumour incidence, increased susceptibility to radiation-induced malignant lymphoma [39]
*Per2* overexpression	Cell cycle arrest, growth inhibition, apoptosis induction	Suppresses breast cancer in vivo [40, 41]
*Per3* deficient		Higher probability of cancer recurrence [7, 62]
*Cry* deficient	Disrupted cell cycle regulation via de-regulation of *Wee-1* and *CyclinD1*	
BMAL1/Era/*Per2* KO	Prevents mammary acinar formation	Facilitates invasion and metastasis [56]
BMAL1 overexpression	Binds to *p53* promoter	Tumour suppression [61]

Circadian mutations covered in this review and their links to cancer. Both epidemiological and experimental data are included, along with possible mechanisms and resultant phenotypes, where known

*KO* knockout, *SNP* single nucleotide polymorphism

## The role of circadian genes in breast cancer

### Altered cell cycle and apoptosis

*Per* mutant mice have revealed a role for circadian gating of cells passing through the cell cycle. Indeed, an altered *Per* gene contributes to malignancy. In humans, there is reduced expression of *Per* genes in both sporadic and familial breast cancer cells compared with normal breast, perhaps occurring via methylation in regions of the *Per* promoter [1]. In mice, *Per2* loss-of-function mutations exhibit higher tumour incidence and show greater susceptibility to radiation-induced malignant lymphoma compared with wild type [39].

**Fig. 2:**
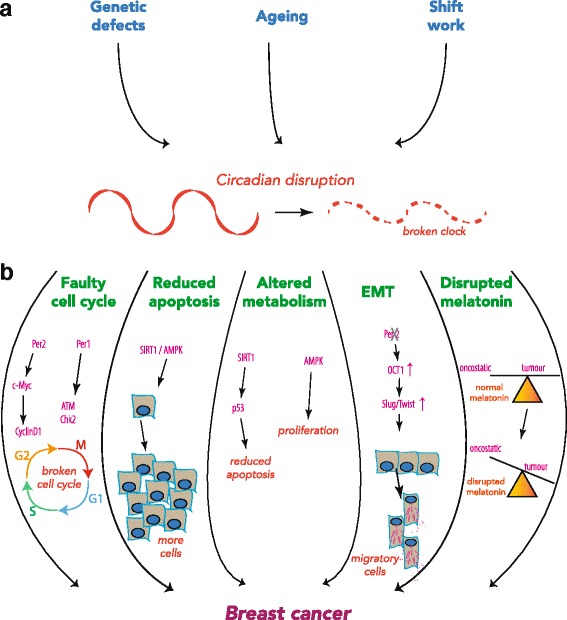
Circadian disruption can drive breast cancer. **a** Intrinsic factors such as genetic defects or ageing, and extrinsic factors such as irregular shift work, can severely disrupt the body’s internal timing system. **b** In turn these factors, either singularly or collectively, link a faulty circadian clock to an increased risk of breast cancer. A faulty clock can directly disrupt the gating of the cell cycle and reduce apoptosis, These effects can also be brought on indirectly through altered metabolism in response to a broken circadian clock. They can also lead to elevated EMT, driving the formation of lethal metastases. A further mechanism that can link to breast cancer is arrhythmic production of melatonin, tipping the balance towards tumorigenesis. *EMT* epithelial–mesenchymal transition

Both PER1 and PER2 also promote apoptosis. Indeed they may suppress breast cancer in vivo by inducing apoptosis [4] (Fig. 2). However, if there is decreased expression of both *Per1* and *Per2* in breast tumours the action of PER as a tumour suppressor becomes reduced. Moreover they can indirectly suppress *c-Myc* transcription by inhibiting E-box-mediated transactivation by BMAL1/Npas2 [32]. Loss of *Per2* leads to decreased apoptosis, and therefore accumulation of damaged cells, by impairing p53 [40]. Consistent with this, *Per2* overexpression in colon cancer results in cell cycle arrest, growth inhibition and apoptosis [41]. Additionally, *Per2* mutants exhibit deregulation of *c-Myc*, and its target cell-cycle genes *CyclinD1* and *Gadd45* [39, 42, 43]. Similarly, inhibition of PER1 alters expression of key cell cycle regulators, by interacting with checkpoint proteins ATM and Chk2, and *Per1* overexpression can reduce proliferation in colon, lung and breast cancer cell lines [41]. Intriguingly in U2OS cells, MYC can regulate the circadian system by binding to E-box elements. High levels of MYC expression dampen the circadian clock and promote proliferation [43].

CRY proteins also impact tumorigenesis via the cell cycle. The cell cycle suppressor WEE-1 is expressed in phase with PER, during times of day when entry to the M phase is suppressed [44]. However, mice deficient in the *Cry* genes have de-regulated *Wee-1* and *CyclinD1*, and therefore exhibit disrupted cell cycle regulation. So far this has been shown in liver cells, but has yet to be explored in breast tumours.

Disruption of PER and CRY therefore causes down-regulation of growth control genes, implying a mechanistic link between the circadian system and cell proliferation. Moreover, these findings indicate that the circadian clock is involved with both cell cycle control and apoptosis.

### Altered cell metabolism

The circadian clock can also initiate and propagate cancer through its effects on cell metabolism (Fig. 2). Metabolic regulators SIRT1 and AMPK are cellular switches that alter cell behaviour according to metabolic state, and both have been linked to the circadian clock [45–47]. SIRT1 is a histone deacetylase that is active under high levels of cellular NAD^+^, and inactive under high levels of NADH [48]. SIRT1 deacetylates p53, inhibiting its activity and reducing apoptosis, which could have implications in cancer [49]. The cellular ratio of NAD^+^ and the deacetylase activity of SIRT1 are both under circadian control [50]. They can feed back into the core clock machinery and thereby regulate the circadian clock [51].

The cellular energy state is reflected in the ratio of AMP + ADP:ATP. When ATP levels are low, AMPK is phosphorylated by an upstream kinase [52]. In its active, phosphorylated state, AMPK regulates many processes, including glucose uptake, mitochondrial biogenesis, cell proliferation and the circadian clock [53]. The cellular clock regulates AMPK activity which in turn induces degradation of the components of the negative arm of the core clock loop, directly phosphorylating *Cry1* and inducing CK1-mediated degradation of *Per2* [47, 54].

Clock disruption could directly impact SIRT1 and AMPK signalling pathways, both of which are crucial in controlling cell proliferation, apoptosis and tumour suppressor pathways. Furthermore, global disruption of circadian behaviour by shift work, jet lag and ageing can affect the feeding schedule of an individual. This could lead to energy imbalances across the circadian cycle and further drive detrimental activity through these signalling pathways.

### Single nucleotide polymorphisms

Breast cancer risk is also associated with single nucleotide polymorphisms (SNPs) in *Npas2* and *Cry2*, as well as *Clock* [36]. Hypermethylation of the *Clock* promoter reduces breast cancer risk, with lower levels of CLOCK in healthy controls. SNPs in *Clock* directly alter the expression of genes linked to cell cycle progression, such as CCL5. Similarly, *Npas2* polymorphisms are associated with increased breast cancer risk. The Ala394Thr SNP alters the NPAS2 protein structure, which interferes with NPAS2/BMAL1 heterodimerization. Women with this SNP are at significantly higher risk of developing the disease ([55], Table 1).

### Link to oestrogen receptor

*Per* gene expression correlates with other genes implicated in breast cancer, for example that encoding the oestrogen receptor (ER). Oestrogen receptor interacts with PER2 and BMAL1 and is integral to the formation of mammary acini, which are the core cellular structures within normal breast [56]. Mammary epithelial cells are highly polarized, and proteins are differentially expressed across the cells [57]. The polarity of the cells and acinar stability are vital for directional milk secretion into alveolar lumens [29]. However, knocking down PER2, BMAL1, or ER prevents acinar formation, perhaps via a feedback loop between oestrogen and the clock [56].

PER1 also influences *ER* transcriptional regulation, while PER2 interacts with ER to suppress oestrogen-mediated transcription of ER target genes [58]. Because *Per* is also induced by oestrogen, there is a feedback loop coupling the circadian clock and the oestrogen pathway. Absent *ER* expression is associated with an aggressive tumour phenotype, and dysregulation of ER-transcriptional activity can equally lead to breast cancer [59]. PER may also act with the breast cancer protein, BRCA1, to regulate *ER* transcription [60].

### Metastasis and epithelial–mesenchymal transition

Low-grade and non-metastasizing tumours maintain functional circadian clocks. In more aggressive carcinomas, however, the coordinated circadian oscillation of clock genes becomes compromised [7]. For example, *Bmal1* expression in the pancreas is significantly reduced in tumours, correlating with cancer severity [61]. In the breast, PER and CRY are associated with better prognosis in ER^+^/HER2^–^ tumours, yet CLOCK and NPAS2 are linked to better prognosis in the more aggressive ER^–^/HER2^–^ tumours. Furthermore, high expression of *Clock*, *Per* and *Cry* is associated with longer metastasis-free survival [7]. In contrast, loss of expression of the *Per3* gene equates with tumours of ER negativity, high histological grade and higher probability of recurrence [62]. Indeed, loss of PER3 and CRY2 co-expression is associated with a higher risk of breast cancer metastasis [7].

While altered levels of clock proteins may affect the tissue structure and contribute to cellular transformation, they also influence an epithelial–mesenchymal transition (EMT) and thereby facilitate invasion and metastasis (Fig. 2). For example, loss of PER2 could directly drive EMT through OCT1. Under normal conditions, PER2 recruits transcriptional co-repressors to OCT1-binding promoters of EMT genes *Twist1*, *Slug* and *Snail*. However, PER2 becomes deregulated in hypoxic, tumour-like conditions, allowing EMT gene expression to be activated [42].

### Summary of the role of the clock in breast cancer

Together these studies show that clock gene defects in mammary epithelium can lead to cell cycle disruption. This disruption causes improper cell division, increased susceptibility to breast cancer and leads to more aggressive tumours. Although there are central roles for circadian genes in normal breast biology, in the following we discuss how disrupting the normal light/dark cycle can also cause disease.

## Shift work, night-time light exposure and breast cancer

Over the last 20 years, epidemiological evidence has corroborated a link between altered clocks and breast cancer. Women who work irregular shift patterns, such as nurses, have a higher frequency of hormone-related breast cancer [63–69], and the higher levels of cancer may be greater for women who started night shifts before a first pregnancy [70]. Elevated breast cancer risk also occurs in women exposed to high levels of ambient light at night [71]. Irregular shift work refers to working a mix of nights and days during a week. In these individuals, the circadian clock has too little time to entrain to the new shift pattern before it is changed again. Notably, however, working consistently at night causes far less disruption to the circadian rhythm.

Mice subjected to a simulated shift-work paradigm have a significantly increased risk of acquiring mammary tumours [72]. By inverting the light cycle every week, mice acquired tumours faster than age-matched littermates housed under normal light/dark cycles. Disruption of sleep patterns, and thereby the internal body clock, of shift workers therefore directly influences their physiology and the rate of cancer development. Moreover, working night shifts three or more times per month elevates the risk of breast cancer in humans. This risk increases with age and with more hours per week of night-shift work [63, 66, 67, 73].

Constant light exposure during the night disturbs circadian pacemaker activity in the mammalian SCN. This ultimately disrupts circadian rhythms throughout the body. One mechanism may be through altered production of the hormone melatonin. Night-time light exposure reduces the rhythmic secretion of melatonin from the pineal gland [74]. This hormone has oncostatic activity in experimental animals with mammary tumours, and also in human breast cancer cells in culture [75] (Fig. 2). Melatonin promotes genomic stability, and may also have anti-invasive and anti-metastatic activity [76]. For example, in endocrine-responsive cancer, melatonin reduces the expression and activity of aromatase, which normally converts testosterone to oestrogen. The oncostatic action of melatonin on hormone-dependent mammary tumours is mainly based on its anti-estrogenic actions, both reducing biosynthesis of oestrogen from androgens and neutralizing the cellular effects of oestrogen. Rats exposed to small amounts of light at night, rather than no light, had disrupted melatonin profiles and faster-growing mammary tumours that were tamoxifen resistant; this could be reversed by melatonin supplementation [77]. In humans, reduction of nocturnal melatonin levels may increase the effects of oestrogen and thereby contribute to breast cancer risk—and indeed, make tumours insensitive to steroid therapy. Epidemiological studies in nurses also reveal that shift work induces a significant increase in circulating oestradiol levels, which could further disrupt mammary oestrogen signalling and thereby promote cancer [78].

Another potential mechanism linking altered light exposure at night-time to breast cancer risk could be via the circadian regulation of micro-RNA (miRNA) expression. Several miRNAs known to be involved with breast cancer risk show fluctuations during the day. For example, the levels of miR-150-5p and miR-133a-3p are dramatically altered by circadian disruption [79]. Some of these miRNAs are interconnected with the expression of proteins known to have roles in breast cancer, such as NFkB and Stat3. The levels of some transcripts that encode proteins involved with cancer are also altered in circadian-disrupted mice [80].

Night-time light exposure can also happen as a result of loss of rhythmic behaviour, such as in SCN-lesioned animals. This both increases the risk of tumour formation and enhances progression of pre-existing tumours. For example, in SCN-ablated mice that are inoculated with implants of osteosarcoma or pancreatic adenocarcinoma, tumours grow two to three times faster than in sham-operated comparators [8]. In the breast, a robust circadian rhythm is associated with slow-growing tumours, while altered rhythms are linked to faster growing tumours [81, 82].

Together the evidence suggests that disrupting circadian clocks via night-time light exposure has an adverse role on tumour progression. This applies to those working irregular shift patterns, although it is still controversial whether there are links in flight attendant jet lag [83] or whether the widespread use of electric lighting at night might contribute to breast cancer [84].

The mechanisms by which altered light exposure contributes to cancer are not yet understood fully. However, the link between circadian disruption and breast cancer may have profound societal importance.

## Conclusion

Tissue-based oscillators within the breast are coordinated by the SCN, and are influenced by photic, endocrine, neural and metabolic cues. Analysis of the strong circadian component within the breast has highlighted its influence on the cell cycle, which impacts on tumour progression and carcinoma aggression.

In-vivo evidence has revealed the role of the circadian molecular clockwork in tumour suppression, highlighting that breast cancer should no longer be treated as a local disorder but rather can result from systemic defects in tissue control.

Mouse models have demonstrated the importance of core clock genes within the breast, whether that is in progenitor cell renewal, tumour incidence, or interaction with the oestrogen pathway. Collectively, this indicates that there is an oncostatic role of some clock genes.

Together with the epidemiological data linking an increased incidence of breast cancer in shift workers, this has serious implications for the demands of modern society. Circadian disruption imposes a major public health issue that has yet to receive the recognition it deserves. Nocturnal lifestyles perturb the normal patterns of circadian behaviour, resulting in detrimental effects on clock-controlled physiological and metabolic pathways. Increased public awareness of the circadian influences on breast cancer risk could enhance lifestyle choices, as well as improve the alignment of physiological systems with the daily body clock.

There is still a fair way to go in characterizing the molecular basis of how the circadian system influences breast cancer, and which of the different genetic types of breast cancer are most influenced by altered clocks. Further delineation of the mechanisms linking clock genes to cancer is needed in order to fully understand how an altered circadian system and how rotating shift work influence the disease. This knowledge will help to improve future therapeutic interventions.

## Abbreviations

BMAL1, Brain and muscle Arnt-like protein-1; CLOCK, Circadian Locomotor Output Cycles Kaput; CRY, Cryptochrome; EMT, epithelial-mesenchymal transition; ER, oestrogen receptor; miRNA, micro-RNA; NPAS2, Neuronal PAS domain protein 2; PER, Period; SCN, suprachiasmatic nucleus; SNP, single nucleotide polymorphism
